# A spatial transcriptomics dataset of pancreas sections in normal glucose tolerance and type 2 diabetic donors

**DOI:** 10.1038/s41597-025-05450-6

**Published:** 2025-09-01

**Authors:** Nick Howell, Zoe Weiss, Lori L. Bonnycastle, Caleb M. Grenko, Davide Randazzo, Christopher H. Dampier, Neelam Sinha, Narisu Narisu, Amy J. Swift, Michael R. Erdos, Leslie G. Biesecker, Francis S. Collins, Catherine C. Robertson, D. Leland Taylor

**Affiliations:** 1 Center for Precision Health Research, National Human Genome Research Institute, National Institutes of Health, Bethesda, MD 20892 USA; 2 Graduate School of Biomedical Sciences, Mayo Clinic, Rochester, MN 55905 USA; 3 Office of Science and Technology, Light Imaging Section, National Institute of Arthritis and Musculoskeletal and Skin Diseases, National Institutes of Health, Bethesda, MD 20892 USA; 4 Laboratory of Pathology, National Cancer Institute, National Institutes of Health, Bethesda, MD 20892 USA; 5 Department of Computational Medicine and Bioinformatics, University of Michigan, Ann Arbor, MI 48109 USA

**Keywords:** Transcriptomics, Type 2 diabetes

## Abstract

Understanding the spatial distribution of gene expression in the pancreas is essential for establishing the molecular basis of pancreatic function in healthy and disease contexts. Recent platforms offer a robust method for quantifying gene expression within a spatial context. Here, we report spatial transcriptomic profiling from pancreas samples obtained from three donors with type 2 diabetes (T2D) and three donors with normal glucose tolerance (NGT). Our analysis identified a major technical challenge: substantial transcript bleed of highly abundant genes (e.g., *INS* and *GCG*) into adjacent tissue regions. We demonstrate that this bleed can be computationally corrected using probabilistic models. Our analysis highlights the importance of incorporating bleed-correction techniques in the preprocessing of spatial transcriptomic profiling data. In summary, this study provides a dataset, methods, and resources to investigate the spatial regulation of gene expression in normal and T2D-affected human pancreas.

## Background & Summary

The pancreas is a dual-purpose organ with exocrine (digestion) and endocrine functions (hormone secretion for blood glucose regulation)^1^. Endocrine cell types and functions are localized to pancreatic islets, mini-organs that control glucose homeostasis through tightly regulated secretion of endocrine hormones including insulin and glucagon^2^. Dysregulated hormone secretion in pancreatic islets is a hallmark of T2D. Spatial mapping of gene expression within the pancreas could improve our understanding of the molecular basis of islet dysfunction leading to T2D.

Historically, studies of spatial trends in gene expression relied on techniques like fluorescence *in situ* hybridization^3^. However, such methods have inherent limitations, such as low throughput capacity and a limited number of markers. Recent advances in spatial transcriptomics have greatly increased the resolution and scale for studying spatial gene expression (reviewed in Rao *et al*.^4^). These spatial transcriptomics technologies—pairing traditional histology with spatial RNA sequencing^5^—have enabled exploration into the spatially resolved transcriptional patterns simultaneously across many genes and tissues^6,7^. To date, few studies using spatial platforms have considered the human pancreas in the normal and diabetic context^8^. To investigate how spatial patterns change between donors with normal glucose tolerance (NGT) and type 2 diabetes (T2D) (Table 1), we generated a dataset of three NGT and three T2D donors, with replication, using the 10x Genomics Visium Spatial Gene Expression v1 platform.

**Table 1:** Donor metadata.

Donor	Status	Age	Sex	BMI	HbA1c	Additional Notes
1	T2D	36	F	33	8.3	Given metformin
2	T2D	56	M	24	5.7	Diagnosed with diabetes 1 year before death. Given insulin for 3 months, metformin for 9 months
3	T2D	47	M	31	6.6	Diagnosed with diabetes 1 year prior to death. Given metformin.
4	NGT	51	M	20	5.2	
5	NGT	43	M	26	5.4	
6	NGT	54	M	25	5.4	

Donor metadata including BMI, HbA1c level, and additional notes about T2D donors.

## Methods

### Source of human pancreas

Human pancreas samples were obtained from Prodo Laboratories (Aliso Viejo, CA). These samples were isolated from cadaverous donors whose organs were consented for research. As per the National Institutes of Health (NIH) Office of Human Subjects Research Protection (OHSRP) policy, tissue obtained from deceased individuals do not fall under the guidelines of human subject research. All experimental protocols performed for this study were approved under NIH guidelines. Approximately 1.5 cm^3^ of pancreas tissue was excised from the tail of each cadaveric pancreas. The tissue was dipped in 1% chlorhexidine and washed with saline to remove contaminants. The tissue was then rinsed in Krebs-Ringer solution containing RNase inhibitor (Sigma R7397) and prepared for cryosectioning by embedding them in optimal cutting temperature (OCT) compound, using isopentane cooled at liquid nitrogen temperature. The frozen tissue was placed into a cryovial cooled on dry ice and stored at −80 °C for up to 72 hours before shipping to our laboratory in dry ice. Upon receipt, we placed the frozen sample in a liquid nitrogen tank for long term storage.

### Sample preparation, fixation, and staining

We embedded frozen pancreatic tissue samples into OCT compound, solidified in an isopentane ice bath to form individual OCT blocks, and maintained at a temperature of −15 °C to −20 °C for processing or at −80 °C for storage. We sectioned tissue OCT blocks into 10 µm sections and placed them onto Visium Spatial Tissue Optimization Slides (PN-3000394) or Visium Spatial Gene Expression Slides (PN-2000233) as recommended by the manufacturer (10x Genomics, Visium Spatial Protocols - Tissue Preparation Guide CG000240). We fixed and stained Visium slides with hematoxylin and eosin (H&E) according to manufacturer’s instructions (10x Genomics, Methanol Fixation, H&E Staining & Imaging for Visium Spatial Protocols CG000160).

### Microscopy for spatial transcriptomics analysis

Following fixation and H&E staining, we imaged the slides using a Leica DMi8 microscope equipped with a CMOS DMC6200 color camera with pixel shift technology (Leica), driven by the Leica LAS X software. We performed image acquisition by employing a HC Plan Apochromatic CS2 10x/0.4 dry lens (Leica) with exposure time of the camera set to 10 ms (consistently for all images) and digital resolution set to 9.2 megapixels (3,840 × 2,400 px, 8 bit). The navigator module of the LAS X software used a tile scanning approach to acquire the entire area of each section, including the fiduciary markers. We captured images as.lef files and exported them as.tif files with lossless compression.

### Optimization of tissue permeabilization conditions

**Fig. 1:**
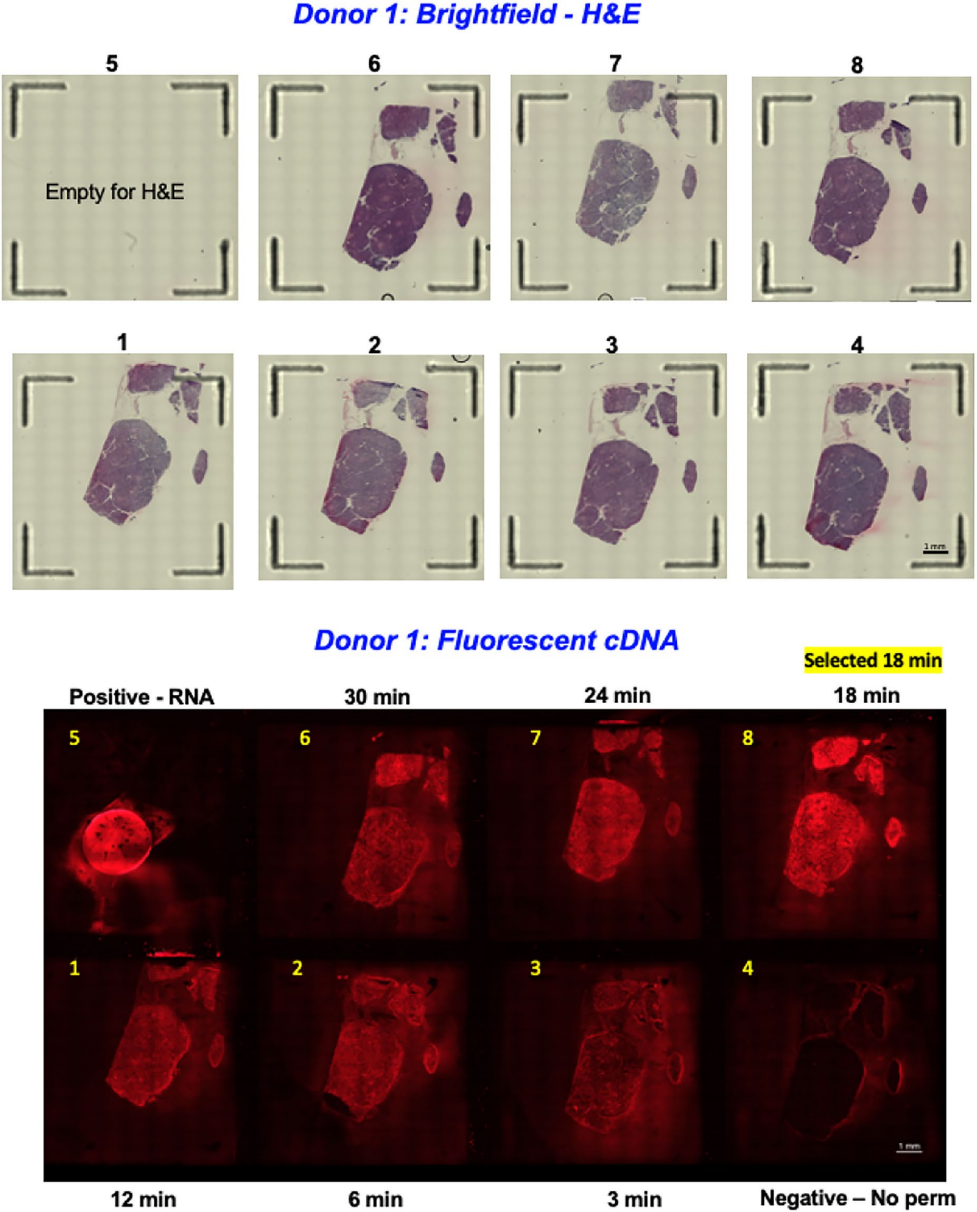
Tissue permeabilization optimization results. Brightfield H&E (top) and fluorescent cDNA (bottom) images from permeabilization time course and fluorescent cDNA synthesis. Image 8 indicates the optimal incubation time of 18 minutes.

After fixation, staining, and imaging, we performed a permeabilization time course followed by fluorescent cDNA synthesis and imaging. We determined an optimal incubation time of 18 minutes for tissue permeabilization (10x Genomics, Visium Spatial Gene Expression Reagent Kits - Tissue Optimization User Guide CG000238) (Fig. 1).

### Spatial RNA capture, library construction, and sequencing

**Fig. 2:**
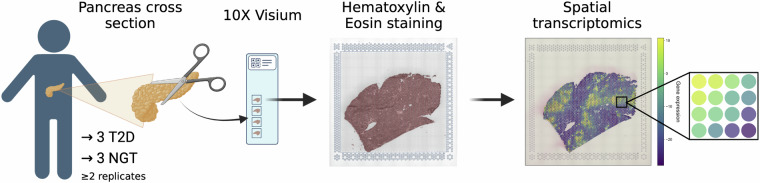
Visium assay workflow. Generation of spatial transcriptomic profiles of human pancreas from normal glucose tolerant (NGT) and type 2 diabetes (T2D) donors. We captured tissue sections with ≥2 replicates onto Visium Spatial Gene Expression slides and performed spatially-resolved RNA sequencing to analyze gene expression patterns within intact tissue sections.

After staining and imaging, we processed Visium slides to generate spatially barcoded cDNA libraries as per the manufacturer’s instructions (10x Genomics, Visium Spatial Gene Expression Reagent Kits User Guide CG000239; Fig. 2). Briefly, we permeabilized the tissue, releasing mRNA for capture by primers on the Visium slide capture areas. Each capture area was 6.5 × 6.5 mm and contained 4,992 spots of spatial barcodes, where each spot is 55 μm in diameter. We reverse transcribed captured mRNAs into cDNA and coupled them to spatial barcodes during second strand synthesis. We transferred spatially barcoded cDNA sequences from the Visium slide to a tube for library construction. We pooled and sequenced dual-indexed libraries on the NovaSeq platform (Illumina, San Diego, CA, USA) with the following read lengths: Read1 + Index1 + Index2 + Read2 (28 + 10 + 10 + 90), where Index1 and Index2 are the sample indices, Read1 contains the 16 bp Spatial Barcode and 12 bp UMI, and Read2 contains the cDNA insert.

## Data Records

We deposited images from the Visium experiments into the Gene Expression Omnibus (GEO) under accession ID GSE264331^9^. We deposited sequencing data (FASTQs from Illumina NovaSeq) and 10x Genomics Visium Space Ranger output (feature, barcode, and raw count matrices as h5 files) as part of the RNA-seq molecular dataset in the database of Genotypes and Phenotypes (dbGaP) under accession ID phs001188.v3.p1^10^. We deposited cloupe files compatible with 10x Genomics’ Loupe Browser software to Zenodo^11^.

Sample naming conventions vary across data repositories. To address this, we have provided a key (Table 2) mapping sample IDs to donor/replicate numbering used in this data descriptor.

**Table 2:** Sample mapping across data repositories.

Donor	Replicate	dbGaP	GEO	Zenodo
1	1	Vis2_S1	Vis2-A	Vis2_S1
	2	Vis2_S2	Vis2-B	Vis2_S2
	3	Vis3_S3	Vis3-C	Vis2_S3
	4	Vis3_S4	Vis3-D	Vis2_S4
2	1	Vis5_S1	Vis5-1A	Vis5_S1a
	2	Vis5_S2	Vis5-1B	Vis5_S1b
3	1	Vis5_S7	Vis5-4A	Vis5_S4a
	2	Vis5_S8	Vis5-4B	Vis5_S4b
4	1	Vis2_S3	Vis2-C	Vis2_S3
	2	Vis2_S4	Vis2-D	Vis2_S4
	3	Vis3_S1	Vis3-A	Vis3_S1
	4	Vis3_S2	Vis3-B	Vis3_S2
5	1	Vis5_S3	Vis5-2A	Vis5_S2a
	2	Vis5_S4	Vis5-2B	Vis5_S2b
6	1	Vis5_S5	Vis5-3A	Vis5_S3a
	2	Vis5_S6	Vis5-3B	Vis5_S3b

## Technical Validation

### Spatial transcriptomic data processing, islet annotation, and quality control procedures

We used Space Ranger (version 2.1.1) with default parameters to process the raw Visium spatial gene expression data and generate gene expression values associated with each spatial location^12^. The gene expression data consist of gene-specific unique molecular identifier (UMI) counts, measured across thousands of 55 μm diameter spots with spot-specific barcodes on 6.5 × 6.5 mm Visium capture areas. We normalized spot UMI counts using scanpy v1.10.1. Sequencing depth varied from spot-to-spot in ST assays. To address this, we normalized spots by total counts per spot and scaled (counts per 10,000 [CP10k]) using scanpy’s normalize_total function.

**Fig. 3:**
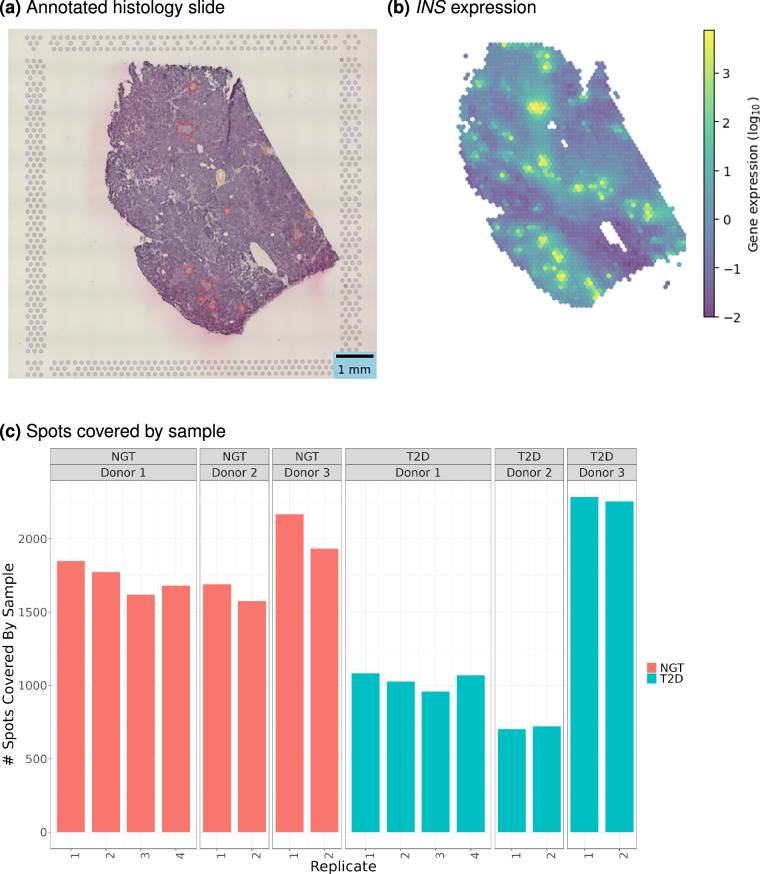
Representative sample expression and quality controls. (**a**) Pathologist annotations overlaid on H&E histology slide, with islet regions circled in red, ducts/ductules in orange, and vascular structures in yellow. (**b**) *INS* expression. (**c**) Number of Visium barcoded spots covered by the tissue sample from each donor.

To verify tissue integrity and accuracy of spatial capture, we conducted a histological review of H&E stained images to identify major histological features, including islets, vasculature, and ducts (Fig. 3a). We compared the identified islet regions to the expression patterns of islet marker genes (e.g., *INS* for beta cells that reside in islets). We found a high overlap of the islet region labelled from H&E image review and the regions with the highest *INS* expression, validating the accurate identification of islets within the pancreatic tissue samples (Fig. 3a,b).

**Fig. 4:**
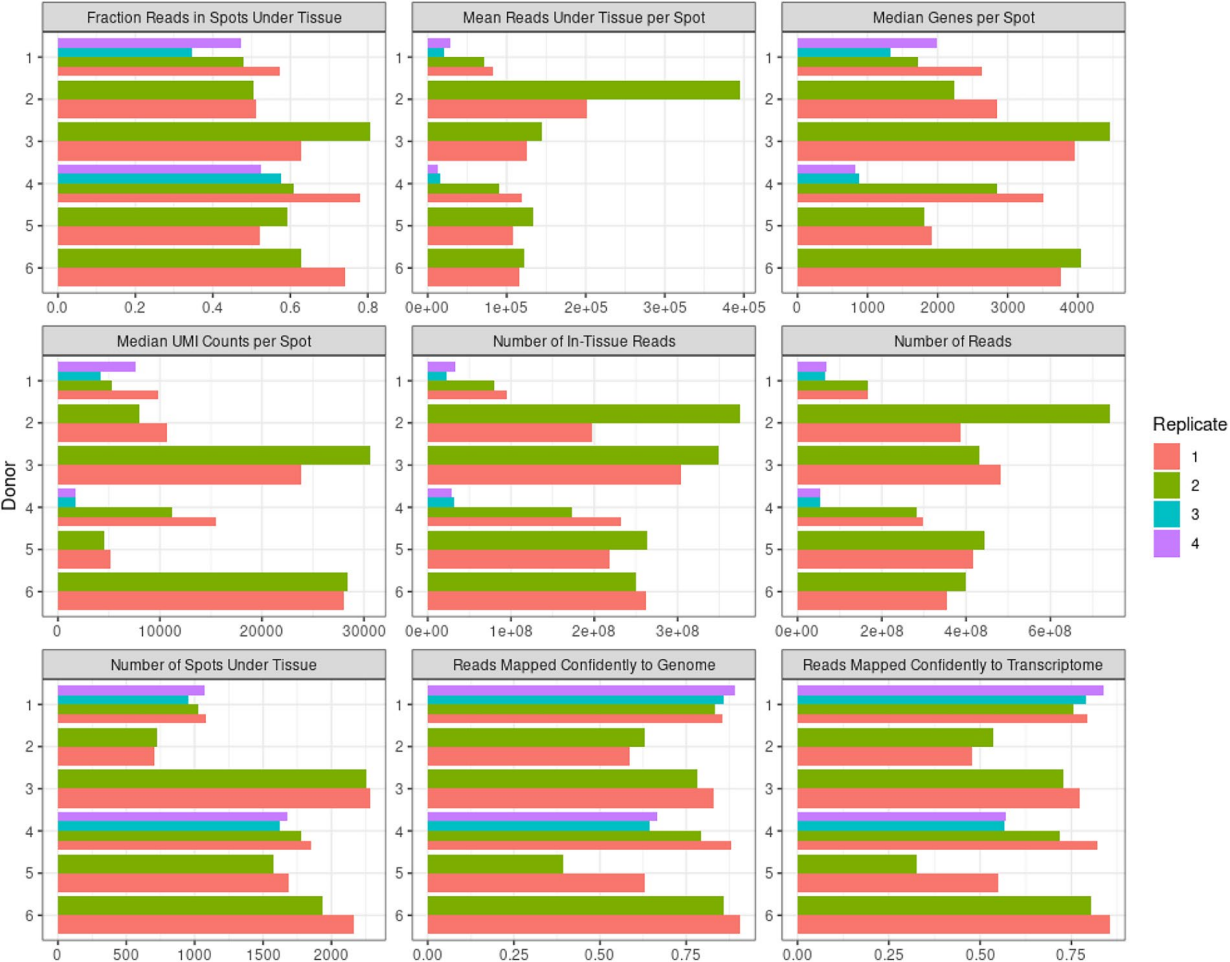
Dataset quality metrics returned by 10X Genomics Space Ranger Pipeline. Quality metrics from 10x Genomics Visium Space Ranger for each processed tissue section from 6 donors, 2–4 replicates each. Replicates are sequential sections or near-sequential sections.

For each sample, our dataset contained two to four tissue slices. We evaluated the number of spots per tissue as an indirect measure of the area of each section (Fig. 3c). We found consistent results across replicates, in line with our expectations as these samples were sequential or near-sequential sections (Fig. 4).

### Identification and cleaning of transcript bleed artifacts

**Fig. 5:**
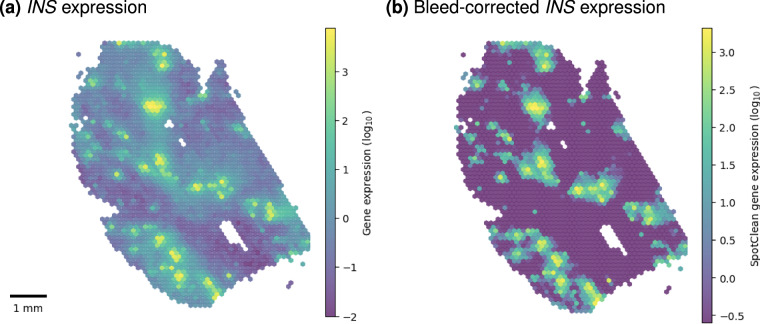
Transcript bleed correction. (**a**) *INS* expression overlaid on H&E histology slide. (**b**) Bleed-corrected *INS* expression.

While the spatial distribution of *INS* expression was largely concordant with manually annotated islets from H&E images (Fig. 3a,b), visual inspection of *INS* expression revealed substantial levels of insulin transcripts across the entire tissue section, including exocrine regions. This observation suggested a degree of transcript diffusion during sample preparation (Fig. 3b). To correct for such transcript bleed, we used a computational approach that uses probabilistic models to adjust spatial gene expression data for transcript bleed (SpotClean^13^ (version 1.4.1)) (Fig. 5).

**Fig. 6:**
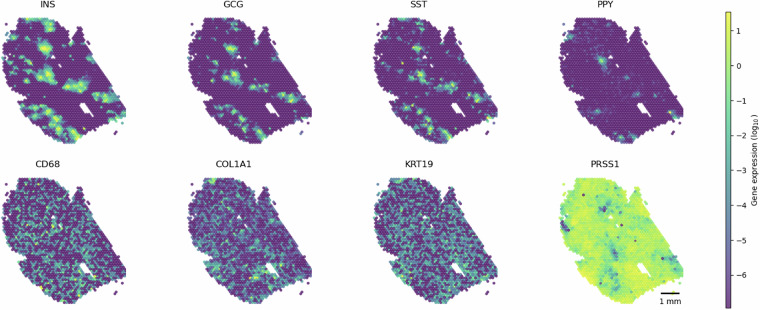
Cell type marker gene expression. Visualization of bleed-corrected gene expression of cell type marker genes. Marker genes include *CD68* (macrophage), *COL1A1* (endothelial), *GCG* (alpha), *INS* (beta), *KRT19* (ductal), *PPY* (gamma), *PRSS1* (acinar), and *SST* (delta)^14^.

After transcript bleed-correction, cell type marker genes for endocrine and exocrine cell types were more cleanly divided into distinct compartments, which more faithfully reflected established understanding of human islets as distinct endocrine cell clusters, delineated by a basement membrane, surrounded by primarily exocrine tissue (dominated by acinar cells) (Fig. 6)^14^. These findings indicated that bleed correction can validate the quality of spatial transcriptomic datasets, and users who access the deposited raw data can replicate this result by following our methods.

### Concluding remarks

In summary, this dataset offers spatial transcriptomic profiles of the human pancreas from both NGT and T2D donors, at 55 μm resolution. Our initial observations of these profiles identified substantial bleed of abundant islet genes, but probabilistic computational correction mitigated this technical artifact effectively.

Limitations of these data include the limited spatial resolution of the Visium Spatial Gene Expression v1 assay and the limited number of donors profiled. The Visium v1 platform spatially barcodes transcripts in a 6.5 × 6.5 mm capture area covered by 4,992 spots, each 55 μm in diameter. At this resolution, data from each spot represented transcriptomes from multiple cells. Recently released assays improve resolution to a near single-cell level^12^.

While exploratory analyses of transcriptional changes in T2D pancreas are a primary application of these data, a larger sample size will be required to overcome donor-to-donor variation when making these comparisons. Having demonstrated the integrity of these data, they may be combined and meta-analyzed with future data sets towards this aim. Moreover, these data alone may be suitable for targeted analyses, including validating the presence or spatial distribution of genes of interest. Notably, since these spatial profiles represent direct transfer of RNA from fresh frozen pancreas and are not dependent on pre-defined human transcriptome probe sets, this resource may be used to detect unannotated and/or non-coding transcripts of interest, which may not be detectable with probe-based RNA assays or protein-based assays (e.g., immunofluorescence imaging) of fixed tissue. Third-party software and frameworks enable continued exploration of spatial patterns across tissues in Visium v1 data. Deep learning models^15^ can improve resolution by predicting transcript counts in inter-spot space on a capture slide. Computational methods, like those provided by the Spatial-eXpression-R (spacexr) library^16,17^, can deconvolute cell types in spots and predict differential expression across spatial axes. Paired with tools like these, the spatially resolved transcriptional profiles of human pancreas presented here may yield additional insights about healthy and T2D pancreas biology.

## Supplementary information

Dataset 1

## Data Availability

The code used for data processing and bleed correction are available at https://github.com/CollinsLabBioComp/publication-visium_preprocessing.
